# Dipeptidyl peptidase-4 inhibitors alleviate cognitive dysfunction in type 2 diabetes mellitus

**DOI:** 10.1186/s12944-023-01985-y

**Published:** 2023-12-11

**Authors:** Jie Meng, Rui Yan, Chen Zhang, Xueyan Bai, Xingsheng Yang, Yu Yang, Tao Feng, Xin Liu

**Affiliations:** 1 Department of Pathology, Beijing TongRen Hospital, Capital Medical University, Beijing, China; 2 State Key Laboratory of Natural and Biomimetic Drugs, School of Pharmaceutical Sciences, Peking University, Beijing, China; 3 Center for Movement Disorders, Department of Neurology, Beijing Tiantan Hospital, Capital Medical University, Beijing, China; 4 National Clinical Research Center for Neurological Diseases, Beijing Tiantan Hospital, Capital Medical University, Beijing, China; 5 Department of Hemotology, Beijing Tiantan Hospital, Capital Medical University, Beijing, China; 6 Department of Cardiology, Beijing Tiantan Hospital, Capital Medical University, Beijing, China; 7 Department of Pharmacy, Beijing Tiantan Hospital, Capital Medical University, Beijing, China

**Keywords:** Dipeptidyl peptidase-4 inhibitors, Cognitive function, MMSE, IADL, Type 2 Diabetes Mellitus

## Abstract

**Background:**

Patients with type 2 diabetes mellitus (T2DM) are commonly at high risk for developing cognitive dysfunction. Antidiabetic agents might be repurposed for targeting cognitive dysfunction in addition to modulation on glucose homeostasis. This study aimed to evaluate the impact of dipeptidyl peptidase-4 inhibitors (DPP-4i) on cognitive function in T2DM.

**Methods:**

PubMed, Embase, Cochrane Library and Web of Science were systematically searched from inception to September 30, 2023. Weighted mean differences were calculated using the Mantel-Haenszel (M-H) fixed or random effects model based on the degree of heterogeneity among studies. Heterogeneity was evaluated using a Chi-squared test and quantified with Higgins I^2^. Sensitivity analysis was performed with the leave-one-out method, and publication bias was evaluated according to Begg’s and Egger’s tests.

**Results:**

Six clinical trials involving 5,178 participants were included in the pooled analysis. Administration of DPP-4i generally correlated with an increase of Mini-Mental State Examination (MMSE) scores (1.09, 95% CI: 0.22 to 1.96). DPP-4i alleviated cognitive impairment in the copying skill subdomain of MMSE (0.26, 95% CI: 0.12 to 0.40). Treatment with DPP-4i also resulted in an increase of Instrumental Activities of Daily Living (IADL) scores (0.82, 95% CI: 0.30 to 1.34). However, DPP-4i produced no significant effects on Barthel Activities of Daily Living (BADL) scores (0.37, 95% CI: -1.26 to 1.99) or other test scores.

**Conclusions:**

DPP-4i treatment favourably improved cognitive function in patients with T2DM. Further trials with larger samples should be performed to confirm these estimates and investigate the association of different DPP-4i with cognitive function among diabetic patients.

**Trial registration in PROSPERO:**

CRD42023430873.

**Supplementary Information:**

The online version contains supplementary material available at 10.1186/s12944-023-01985-y.

## Introduction

The number of patients with diabetes is increasing yearly worldwide. According to a report by the World Health Organization, the number of diabetic patients would reach 366 million in 2030 [1]. Among them, approximately 90% of patients were diagnosed with type 2 diabetes mellitus (T2DM) [2]. A previous study explored the association of social risk factors with diabetes, demonstrating that patients’ educational levels and ethnicity contributed more to the development of diabetes than other factors [3]. The social factor of stress is also linked with the onset of T2DM [4]. Both modifiable (for example, obesity, smoking, diet, insufficient physical activity, excessive alcohol consumption and even unhealthy sleep) and nonmodifiable risk factors (for example, age, sex and ethnicity) contribute to the development of T2DM [5]. Among them, aging is a key factor leading to an increasing number of diabetic individuals [6]. A similar increasing trend has also been observed in related neurodegenerative diseases. Cognitive dysfunction is one of the manifestations of diabetes. Diabetes has been identified as a risk factor for cognitive dysfunction, including cognitive decline, mild cognitive impairment (MCI) and dementia [7]. Diabetic patients are likely to develop neurodegenerative complications ranging from cognitive impairment to dementia [8]. Additionally, patients with neurological diseases are at a high risk for developing diabetes [9, 10]. Accumulating evidences have demonstrated a positive correlation between diabetes and cognitive dysfunction [11–14]. Furthermore, multiple cognitive domains, including execution and spatial skills, are affected during the development of cognitive dysfunction in diabetic state [15]. Although numerous efforts have been made to alleviate cognitive dysfunction, no effective agents could arrest or reverse the process underlying cognitive impairment.

An increased understanding of the common pathology between diabetes and cognitive disorders has led to a focus on the therapeutic potential of antidiabetic agents for treating cognitive dysfunction [16–20]. High levels of glycosylated haemoglobin (HbA1c) have been found to positively correlate with an increased risk of cognitive dysfunction [21]. Antidiabetic regimens are expected to be repurposed for treating cognitive impairment in addition to modulation of glucose homeostasis. In a large observational study involving 176,250 participants, antidiabetic agents, including metformin, dipeptidyl peptidase-4 inhibitor (DPP-4i), glucagon-like peptide-1 (GLP-1) analogues and sodium glucose cotransporter 2 inhibitors (SGLT2i), significantly reduced the risk of dementia in T2DM [22]. Among these antidiabetic regimens, DPP-4i have served as oral agents by interacting with DPP-4 since 2006 [23]. A previous study demonstrated that high concentrations of DPP-4 or enhanced DPP-4 activity might be positively linked with cognitive impairment in elderly participants with T2DM [24]. DPP-4 affects cognitive function in diabetic patients through multiple signalling pathways involving inflammatory reactions, oxidative stress and mitochondrial dysfunction [25]. Inhibition of DPP-4 potentially produced an effect on cognitive function in addition to the action on GLP-1 [26]. In fact, studies have indicated that DPP-4i presented pleiotropic effects in addition to their antihyperglycaemic actions. For example, sitagliptin significantly alleviated memory impairment by promoting neurogenesis and reducing oxidative stress [27]. An improvement of the Mini-Mental State Examination (MMSE) score has been observed in diabetic patients undergoing treatment with DPP-4i [28]. Compared to nonusers, DPP-4i users showed a slower cognitive decline based on MMSE scores [29]. Linagliptin was also demonstrated to slow the progression of premature aging by enhancing cerebral blood flow in gene-modified mouse models [30]. In contrast, other studies presented no beneficial effects on cognitive function during DPP-4i treatment. Furthermore, DPP-4i were even suggested to deteriorate cognitive function and produce neurodegenerative toxicity in diabetic patients. For example, sitagliptin increased tau phosphorylation in the hippocampus of diabetic rats, leading to a caution on the administration of sitagliptin in treating Alzheimer’s disease (AD) [31].

To date, no definite conclusion has been achieved regarding the potential effects of DPP-4i on improving cognitive dysfunction in T2DM [32]. This meta-analysis was designed to ascertain whether DPP-4i could improve or deteriorate cognitive function in T2DM.

## Methods

### Search strategy

This meta-analysis was reported in accordance with the Preferred Reporting Items for Systematic Reviews and Meta-Analyses (PRISMA) statement [33]. The protocol was registered in the PROSPERO system (CRD42023430873). PubMed, Embase, Cochrane Library and Web of Science were systematically searched from inception to September 30, 2023. Key words combined with free words were used to perform the search. The index words mainly included (dipeptidyl peptidase-4 inhibitors) AND (Diabetes Mellitus, Type 2) AND (cognitive function).

### Study selection

Relevant studies were screened by two reviewers. Disagreements were resolved by consulting a third researcher until consensus was reached. Eligible trials evaluating the impact of DPP-4i on MMSE scores, Barthel activities of daily living (BADL) scores, instrumental activities of daily living (IADL) scores and scores on other tests were recorded. The inclusion criteria were as follows: (i) trials evaluating the effect of DPP-4i on cognitive function and related scores; (ii) presented sufficient information on the pre- and post-treatment MMSE, BADL, IADL scores and other assessment scores or provided a difference score; and (iii) enrolled patients with T2DM. Animal studies, letters, case reports, meeting abstracts and narrative reviews were excluded. Studies without sufficient information on pre- or posttreatment-related cognitive scores were also excluded. The inclusion and exclusion criteria were objectively evaluated by two reviewers.

### Data extraction

The following data were extracted by two reviewers: first author’s name, publication year, study location, gender ratio, number of participants in the DPP-4i and control groups, mean age, body mass index (BMI), treatment duration, diabetes duration, baseline levels of HbA1c, and baseline and post-treatment scores of cognitive tests. The longest therapy duration information was extracted if multiple follow-ups were reported in the same study. Parameters of cognitive function included MMSE, Verbal Fluency Test (VFT) and Trial Making Test (TMT). The comprehensive geriatric scales included the BADL, IADL, Center for Epidemiologic Studies Depression Scale (CES-D), Global Deterioration Scale (GDS) and Mini Nutritional Assessment (MNA). All the parameters were extracted as an absolute value pre- and post-treatment in T2DM. The ratio of TMT was recorded and pooled to analysis.

### Quality assessment

The quality of randomized controlled trials (RCTs) was evaluated according to Cochrane Handbook for Systematic Reviews of Interventions as our previous study [34]. The following domains were assessed: random sequence generation; allocation concealment; blinding of participants, personnel, and outcome assessment; incomplete outcome data; selective outcome reporting and other potential sources of bias. According to the Cochrane criteria, a judgement of ‘yes’ indicates a low risk of bias, while ‘no’ indicates a high risk of bias. A judgement of ‘unclear’ indicated an unknown risk of bias. The Newcastle‒Ottawa Scale was used to assess the quality of non-RCTs included [35].

### Quantitative data synthesis

Meta-analysis was performed to examine cognitive test scores. Weighted mean difference (WMD) and 95% confidence interval (CI) were calculated to examine test scores among T2DM participants. A fixed effects or random effects model was used based on the heterogeneity, which was quantified with the *I*^*2*^ index. Pooled analysis was considered to be statistically significant when *P* value was < 0.05. Sensitivity analysis was performed using the leave-one-out method to examine the influence of each individual study. Publication bias was also examined by Begg’s test and Egger’s test if there were at least five studies reporting an outcome. Statistical analysis was performed with Review Manager and STATA 12.0 software.

## Results

### Characteristics of the included studies

**Fig. 1:**
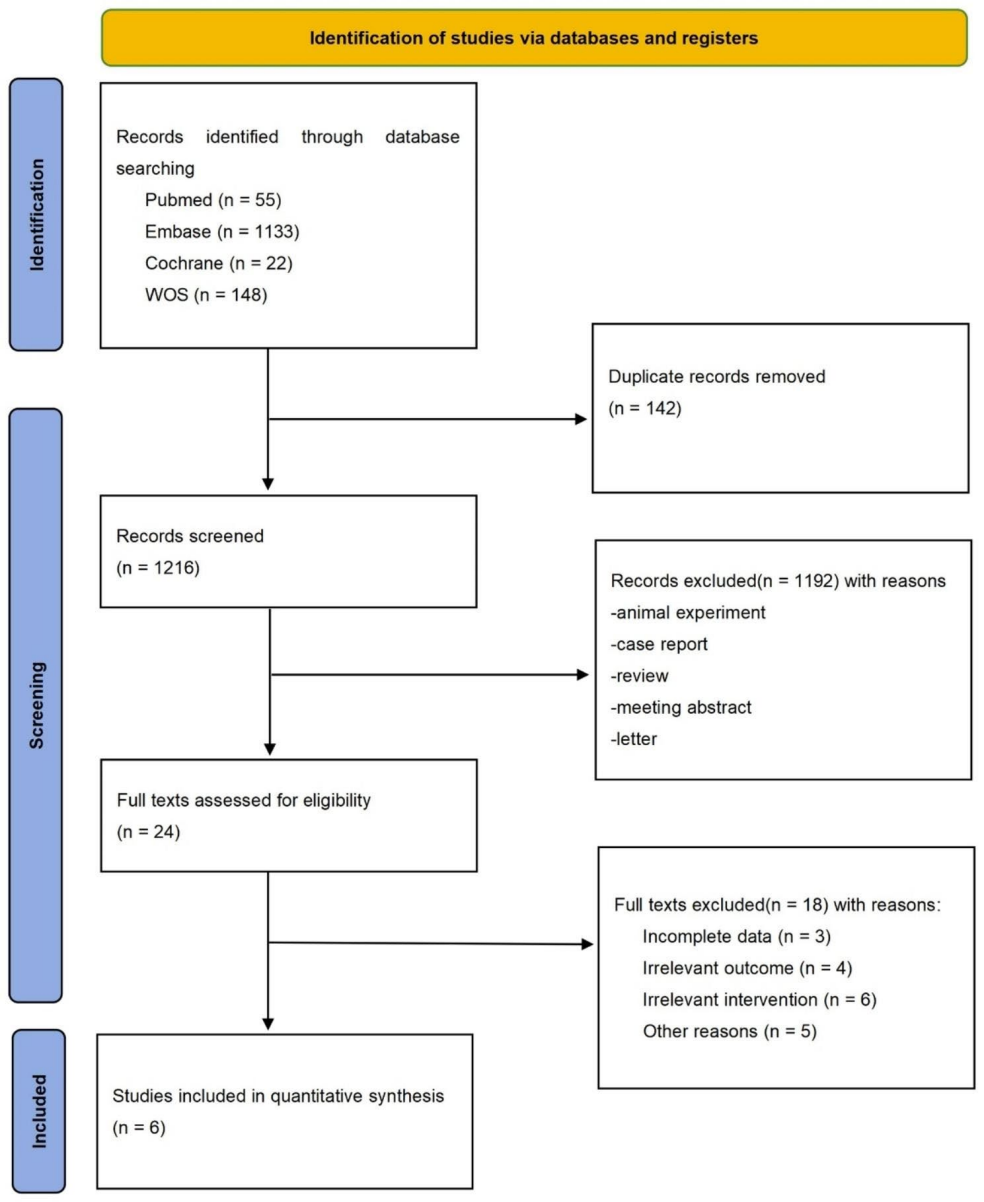
PRISMA flow chart for study selection

After a detailed literature search, six studies ultimately met the inclusion criteria [36–41]. The search process is demonstrated in Fig. 1. For the MMSE, 2,650 patients were placed in the DPP-4i group, and 2,528 patients were placed in the control arm. Two studies lasted less than 1 year (ranging from 1 to 6 months), and four studies lasted more than 1 year (ranging from 13 to 26 months). Two studies had a sample size larger than 1000. The baseline characteristics of included studies were shown in Table 1. Participants received DPP-4i treatment with linagliptin, vildagliptin and sitagliptin. Cognitive function was evaluated by the scores of MMSE, VFT and TMT. The comprehensive geriatric scales included tests of BADL, IADL, CES-D, GDS and MNA. Overall, the quality of the identified trials was moderate (see supplementary Tables 1 and supplementary Table 2).

**Table 1:** Demographic characteristics of the studies included

Study/year	Location	Treatment arm (n)	Sex (male/female)	Duration of follow-up (months)	Age (years)	Duration of diabetes (years)	BMI (kg/m^2^)	HbA1c (%)	MMSE score (0[worst]-30[best])
Isik,2017 [36]	Turkey	sita: 43 met: 69 insu: 22	sita: 47/57 met + insu: 35/66	6	77.05 ± 8.50 77.12 ± 7.87 77.61 ± 9.40	13.38 ± 8.5 12.95 ± 4.99 15.66 ± 6.97	28.86 ± 4.21 29.78 ± 4.71 28.33 ± 5.24	7.29 ± 1.49 6.90 ± 1.29 7.50 ± 1.19	22.73 ± 5.64 23.55 ± 5.38 23.50 ± 4.03
Borzi,2019 [37]	Italy	vild + met: 30 met: 30	vild + met: 14/16 met:13/17	6	77.67 ± 8.23 75.46 ± 7.89	16.72 ± 5.81 15.49 ± 6.83	29.55 ± 5.92 28.48 ± 6.69	8.02 ± 1.27 7.09 ± 1.21	21.00 ± 1.41 20.77 ± 1.22
Biessels,2019 [38]	Netherland	lina: 800 pla: 745	lina: 503/297 pla: 501/244	30	67.8 ± 8.3 67.7 ± 8.0	14.8 ± 9.2 15.4 ± 9.4	32.5 ± 5.1 32.8 ± 5.3	7.8 ± 0.9 7.8 ± 0.9	28.3 ± 1.7 28.2 ± 1.8
Xue, 2020 [39]	China	dpp4i: 30 sulfo: 30	dpp4i: 17/13 sulfo: 14/16	6	68.5 ± 7.1 67.4 ± 5.9	8.17 ± 3.05 8.97 ± 2.65	25.71 ± 3.76 25.59 ± 4.28	8.56 ± 1.25 8.86 ± 3.17	25.42 ± 1.22 25.37 ± 1.16
Bulut,2020 [40]	Turkey	vild: 43 pla: 35 con: 52	vild: 18/25 pla + con: 20/32	6	74.4 ± 7.9 79.7 ± 4.8 74.2 ± 7.8	18.4 ± 9.9 11.3 ± 7.6 16.4 ± 10.6	29.3 ± 5.1 28.5 ± 4.2 29.1 ± 4.5	7.63 ± 1.37 6.01 ± 0.49 7.12 ± 1.57	21.81 ± 6.16 24.94 ± 4.31 26.04 ± 5.04
Biessels,2021 [41]	Netherland	lina: 1618 glim: 1545	lina: 1002/616 glim: 958/587	40	64.4 ± 9.1 64.4 ± 9.3	7.7 ± 6.2 7.4 ± 5.9	30.8 ± 5.0 30.7 ± 4.9	7.1 ± 0.5 7.1 ± 0.6	28.5 ± 1.7 28.5 ± 1.7

Values are expressed as the mean ± SD. Abbreviations: n: number of participants per group; HbA1c: glycated haemoglobin; sita: sitagliptin; vild: vildagliptin; pla: placebo; con: conventional treatment; met: metformin; insu: insulin; sulfo: sulfonylurea; lina: linagliptin; gli: glimepiride; MMSE: Mini-Mental State Examination

### Effect of DPP-4i treatment on cognitive function

**Fig. 2:**
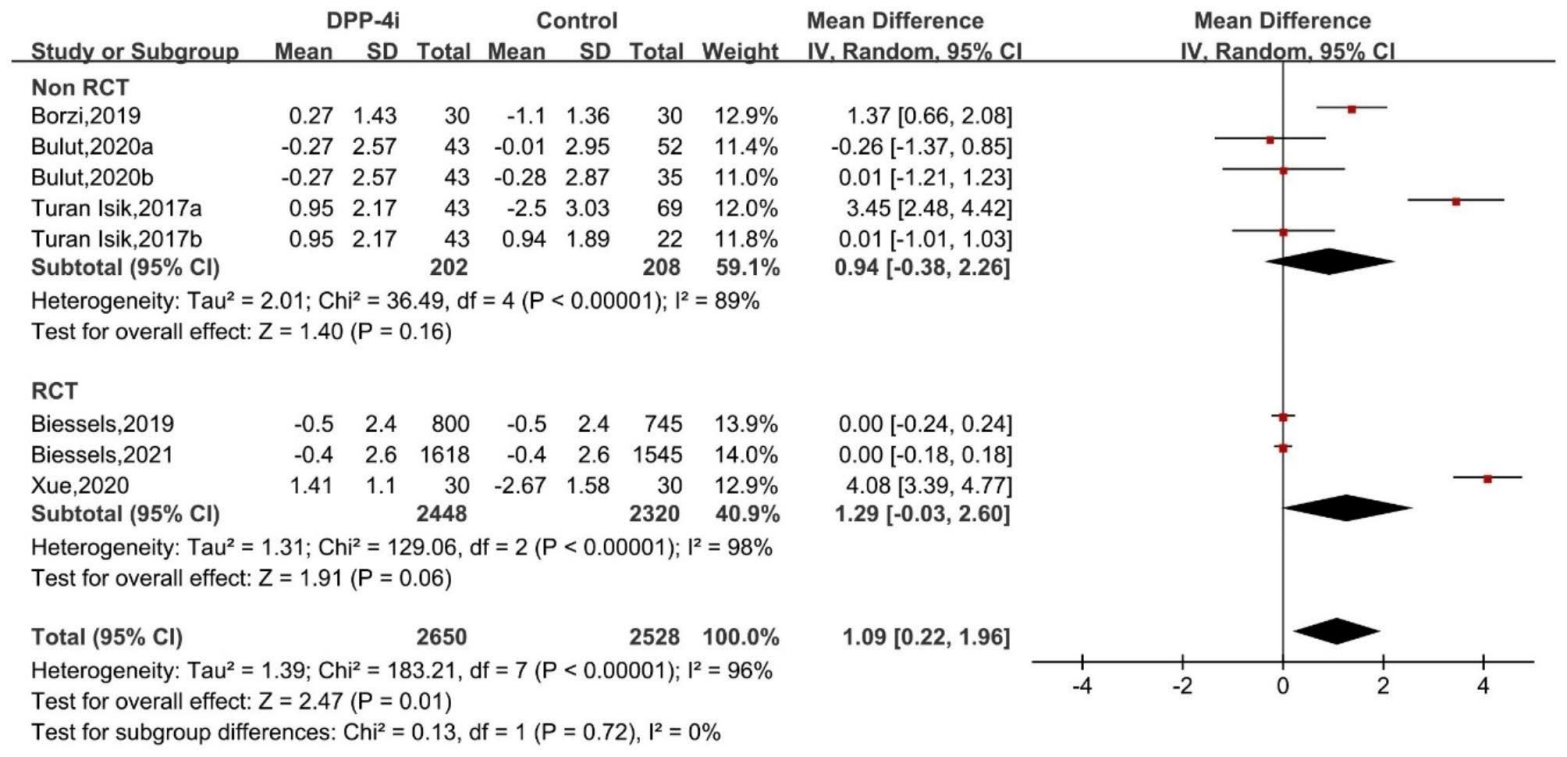
Forest plot for the analysis of the impact of DDP-4i on MMSE total and subdomain scores

**Fig. 3:**
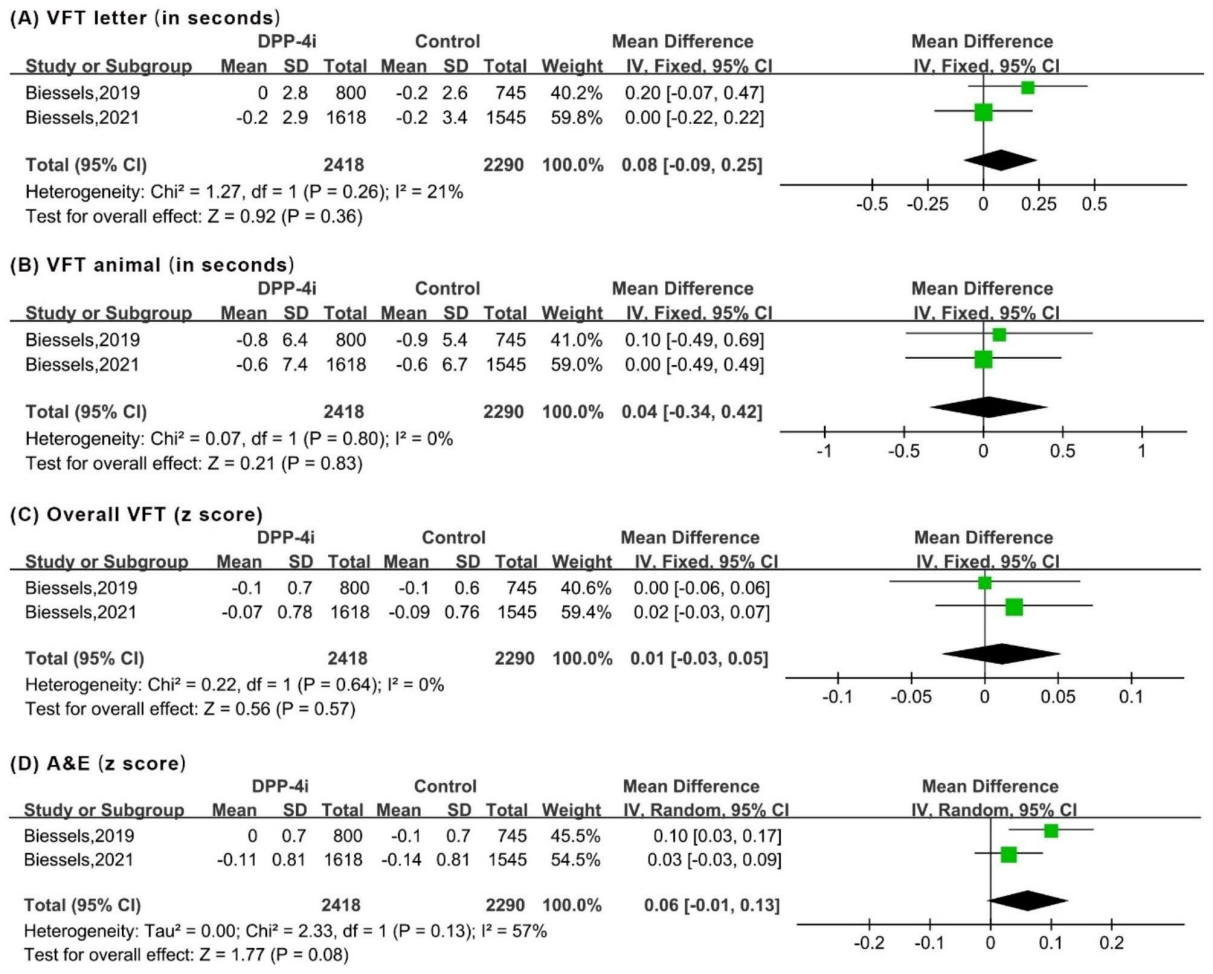
Forest plot for the analysis of the impact of DDP-4i on the VFT score

**Fig. 4:**
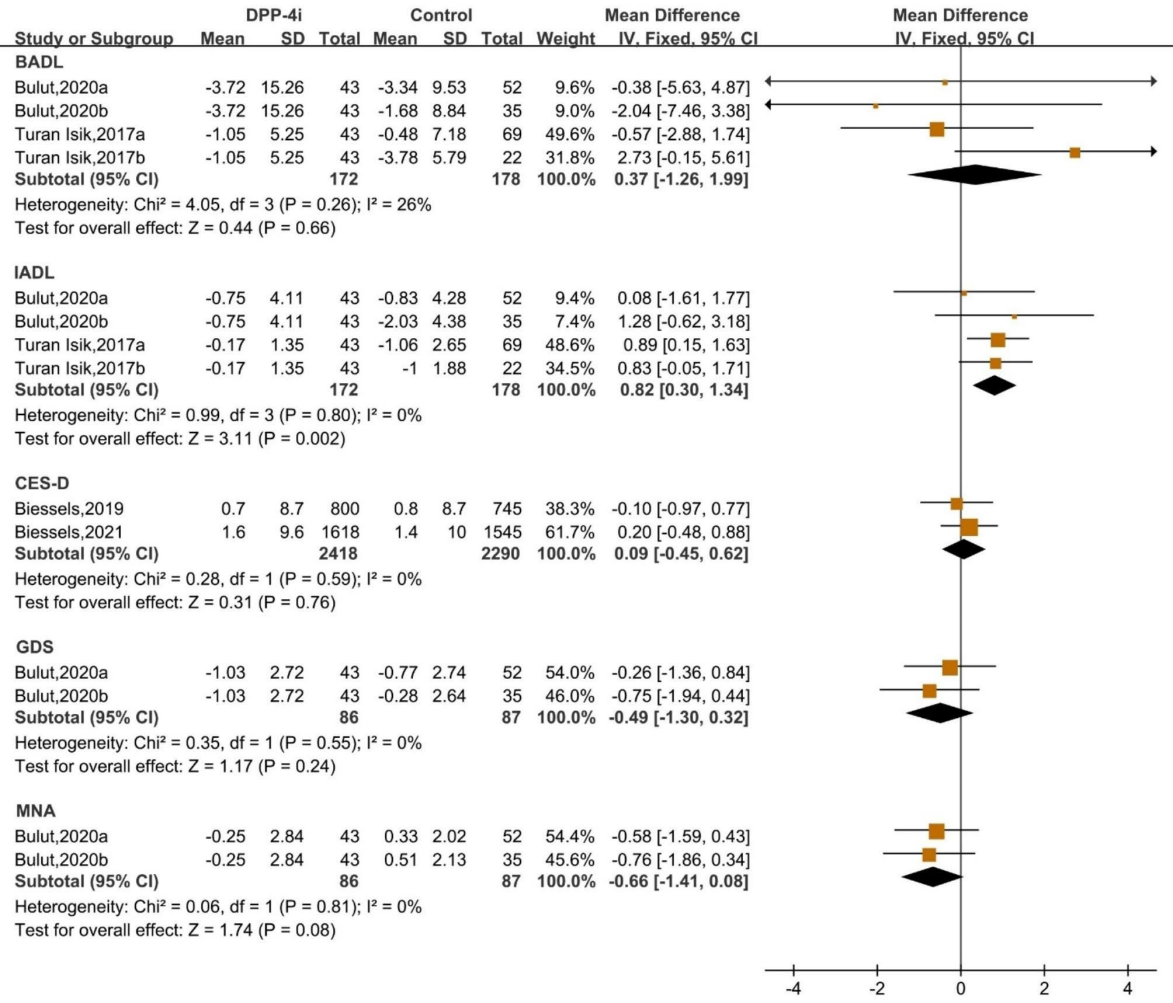
Forest plot for the analysis of the impact of DDP-4i on the TMT score

Meta-analysis suggested that DPP-4i produced an improvement of the overall MMSE score (WMD: 1.09, 95% CI: 0.22 to 1.96). Regarding the subdomains of MMSE, significant improvements were observed in copying skills (WMD: 0.26, 95% CI: 0.12 to 0.40). However, DPP-4i produced no significant effects on other subdomains, including temporal orientation, records, language, attention and recall (Fig. 2). Similarly, for VFT and TMT, DPP-4i treatment showed no significant improvements, as indicated by outcomes of VFT letter 60 (in seconds), A&E (z score), VFT animals 60 (in seconds), overall VFT 60 (z score), TMT-A (in seconds), TMT-B (in seconds) and TMT ratio (Figs. 3 and 4).

### Effect of DPP-4i treatment on comprehensive geriatric assessment

**Fig. 5:**
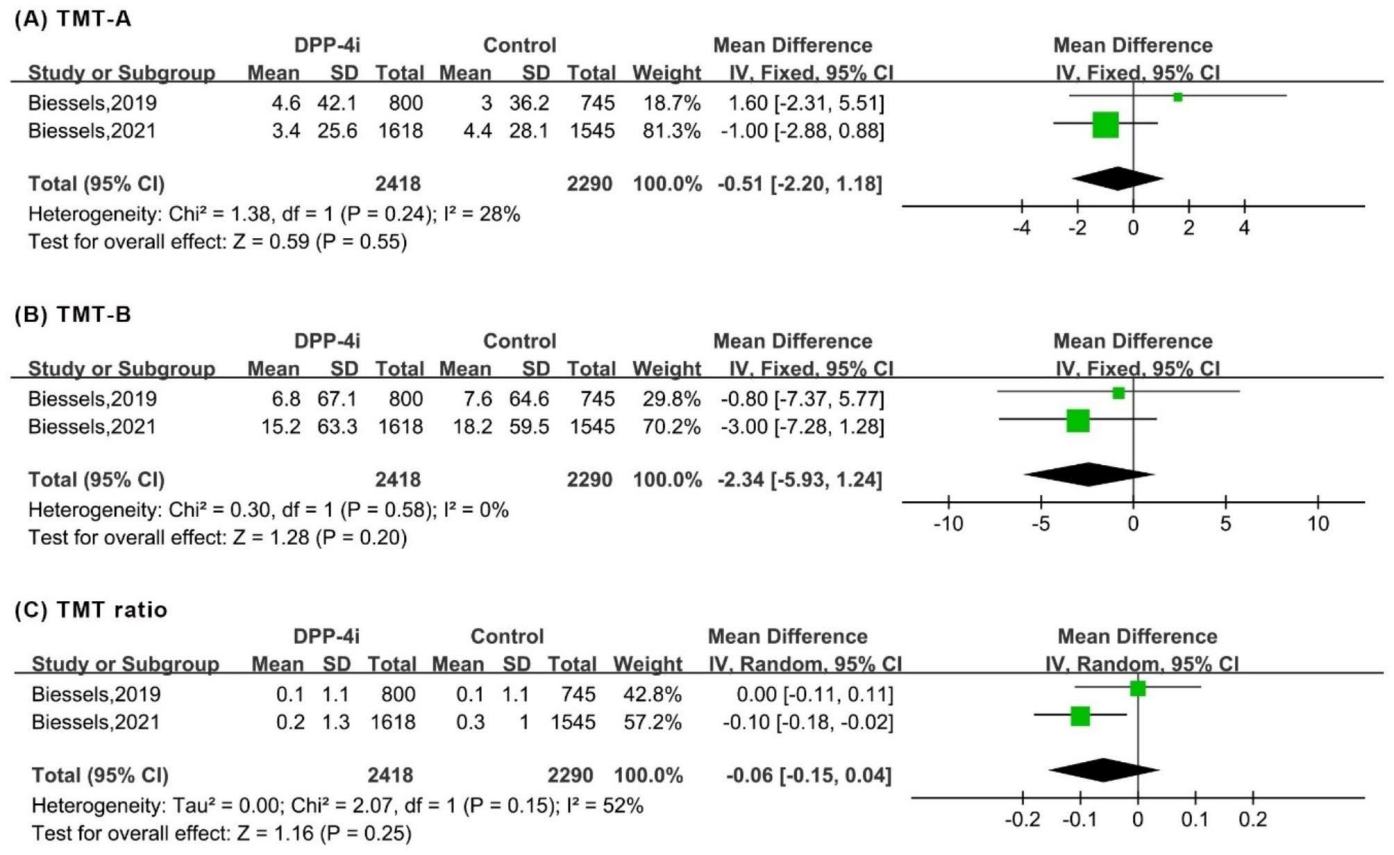
Forest plot for the analysis of the impact of DDP-4i on the comprehensive geriatric assessment

DPP-4i treatment was found to produce a favourable effect on comprehensive geriatric assessment, as shown by a significant change of IADL score (WMD: 0.82, 95% CI: 0.30 to 1.34). DPP-4i treatment produced no significant effect on BADL score (WMD: 0.37, 95% CI: -1.26 to 1.99). Similarly, CES-D, GDS and MNA scores were not modulated during DPP-4i treatment (Fig. 5).

### Sensitivity test and publication bias evaluation

Sensitivity analysis revealed that the beneficial effect of DPP-4i on MMSE scores was stable (WMD: 1.09, 95% CI: 0.22 to 1.96, N = 8 studies, heterogeneity *P* = 0.014) (Supplementary Fig. 1). This finding indicated that a significant difference across studies is an overall effect from all studies included. Furthermore, no publication bias of cognitive function was detected by Begg’s test (*P* = 0.62) or Egger’s test (*P* = 0.15) (Supplementary Fig. 2).

## Discussion

In the current meta-analysis, the pooled estimates of MMSE and IADL scores confirmed an improvement of cognitive function in diabetic patients receiving DPP-4i treatment. DPP-4i could be potentially used for diabetes-related cognitive disorders in clinical practice.

Six studies were identified to evaluate the impact of DPP-4i on cognitive function in T2DM. In the study by Isik et al., elderly diabetic patients with or without cognitive dysfunction were administered with sitagliptin. During a follow-up of 6 months, sitagliptin increased MMSE scores in patients with AD compared to metformin treatment [36]. In addition, concomitant administration of vildagliptin with conventional antidiabetic treatment also improved cognitive function by targeting copying subdomain in older diabetic patients [40]. This improving effect on cognition might come from an increase of brain GLP-1 levels. Further study demonstrated that vildagliptin prevented the reduction of MMSE in elderly diabetic patients on the basis of metformin treatment [37]. Two studies by Biessels et al. evaluated the impact of linagliptin on cognition in diabetic patients, and no decline of cognitive performance were observed when compared to control group [38, 41]. It is interesting to find that fewer patients in linagliptin treatment group developed depressive manifestations. DPP-4i also improved cognitive ability in elderly diabetic patients with post-stroke MCI, which might be associated with a reduction of Aβ expression and inflammatory response [39].

The exact mechanism through which DPP-4i modulated cognitive function remains unclear. Numerous studies have revealed that multiple common pathologies underlie diabetes and cognitive decline, involving insulin resistance (IR), mitochondrial disorders, oxidative stress, glucose fluctuation and inflammatory response [27, 42–45]. DPP-4i might modulate cognitive function by targeting one or more of the signalling pathways above in a direct or indirect manner. IR is commonly present in diabetic patients and serves as an important risk factor for developing cognitive dysfunction. Additionally, neuroimaging results revealed subtle cerebral abnormalities in the insulin-resistant brain [46]. Upregulation of DPP-4 expression in the liver was usually accompanied by IR [47], while insulin sensitivity could be significantly improved when DPP-4 expression was downregulated [48]. Furthermore, DPP-4i users demonstrated an improved insulin sensitivity and secreted more insulin from beta-cells, a phenomenon partially correlated with a reduction of DPP-4 activity [49]. Additionally, vildagliptin treatment effectively improved neuronal insulin sensitivity and prevented brain mitochondrial dysfunction, thus contributing to the alleviation of cognitive impairment induced by a high-fat diet [50].

In a previous study, DPP-4 induced mitochondrial dysfunction and reduced cognitive function in T2DM, a process mediated by the protease-activated receptor 2 (PAR2)-dependent signalling pathway in the hippocampus. A reversal of mitochondrial dysfunction was achieved when *DPP-4* gene was silenced, followed by an improvement of cognitive dysfunction in db/db mice [51]. An upregulation of DPP-4 expression had been suggested to be related to cognitive impairment via an overload of mitochondrial calcium, while cognitive impairment was alleviated after downregulation of DPP-4 expression [52]. The improvement in mitochondrial function might be linked to an alleviation of oxidative stress and inflammatory response in DPP-4i-treated subjects. Furthermore, enhanced DPP-4 activity increased oxidative stress and activated inflammatory response, partially contributing to cognitive impairment in T2DM [53]. Linagliptin favourably alleviated cognitive dysfunction through downregulating NADPH expression in streptozotocin-induced diabetic models, a process mediated by a reduction of oxidative stress [54]. Sitagliptin presented an anti-inflammatory effect through activating NF-E2-related factor 2 (Nrf2), thus leading to an alleviation of excessive autophagy in lipopolysaccharide (LPS)-induced inflammatory models [55]. Additionally, DPP-4i restricted diabetes-associated cognitive deficits by modulating neuroinflammatory indicators of caveolin 1 (Cav 1) and brain-derived neurotrophic factor (BDNF), thus reducing inflammatory response in the brain [56]. A previous study also explored the association of glucose fluctuation with cognitive function. Patients with glucose fluctuation commonly demonstrated an impaired performance of TMT-B and VFT [45].

The efficacy of DPP-4i on cognitive function was also found to be indirectly associated with a reduction of DPP-4 activity [44]. Plasma levels of DPP-4 substrates, including GLP-1, stromal derived factor 1α (SDF-1α) and brain natriuretic peptide (BNP), were increased during DPP-4i treatment. These substrates were either activated or inactivated by DPP-4 to partially modulate related signalling pathways involving inflammation or oxidative stress, thus facilitating cognitive impairment in diabetic conditions. Furthermore, substrates of SDF-1 and GLP-1 have also been suggested to provide neuroprotection in AD models [57]. DPP-4i prolonged the enzymatic degradation mediated by DPP-4, thus enhancing the neuroprotective effect of those substrates [58]. Additionally, DPP-4i have been shown to improve cognitive function by increasing concentrations of adiponectin receptor 1 and hypothalamic acetylcholine in T2DM [59]. In a recent study, DPP-4i increased the recruitment of circulating brain stem cells towards lesion sites, thus resulting in an increase of synaptic plasticity and neurogenesis. This suggested that DPP-4i might improve cognitive function by promoting hippocampal neurogenesis in AD [60].

In the current pooled analysis, DPP-4i were shown to improve MMSE scores in T2DM patients. The pooled estimate was consistent with a study performed by Rizzo et al., in which MMSE and composite cognitive function scores were also significantly improved in elderly diabetic patients with MCI during DPP-4i treatment [61]. Similarly, vildagliptin was shown to alleviate diabetes-associated cognitive impairment by reducing apoptosis-related protein levels in the hippocampus [62]. A beneficial effect of DPP-4i on cognitive function was also found in an insulin-resistant context. For example, cognitive decline was restored after vildagliptin administration in insulin-resistant obese subjects [63]. Further studies should be performed to fully explore the effect of DPP-4i on cognitive function in T2DM. In addition to an improvement of MMSE scores, DPP-4i restored neuroplasticity in patients with T2DM, while no favourable effects on odour detection and olfactory memory were observed [64]. Notably, the improved neurological function might not correlate with glycaemic control from DPP-4i treatment. Indeed, the effect of DPP-4i on cognitive function might be common in multiple neurodegenerative diseases. DPP-4i have also been shown to reverse amyloid deposition in AD patients with cognitive impairment [65]. The protective effect might potentially correlate with an enhanced glucose uptake, an action mediated by GLP-1 [66]. An animal study also showed that linagliptin ameliorated cognitive decline in a tauopathy mouse model [67]. In contrast, other studies showed that DPP-4i exerted no significant effect on modulating cognitive function in T2DM. For example, saxagliptin provided no neuroprotection or improvement of cognitive or motor function in 6-hydroxydopamine-induced Parkinson’s disease (PD) models [68]. Inconsistent results above might be attributed to the heterogeneity of models and contexts.

Sensitivity analysis revealed some heterogeneity in the outcomes of the identified studies. The heterogeneous result might come from a physiological or pathological effect or a lack of significant neurological changes during DPP-4i treatment [69]. Different patient ages and combined drug use also produced an effect on cognitive function during DPP-4i treatment. These multiple results on cognition might also result from different test measures used at baseline among these studies. Meta-regression was not conducted owing to the limited number of included studies. The correlation between DPP-4i treatment and cognitive function should be further explored considering variables including races or disease-related complications at baseline. DPP-4i improved pancreatic β-cell function in both fasting and postprandial states in T2DM. The reversal of hippocampal IR mediated by alogliptin had been reported to improve cognitive function in an AD model [70]. Further studies needed to be performed to analyse the association of insulin secretion with cognitive function in DPP-4i users.

The current meta-analysis also revealed that DPP-4i had a significant effect on improving IADL scores. Although IADL is mainly an evaluation tool of daily living ability, this tool is also suggested to reflect cognitive function indirectly [36, 40]. Due to the limited studies identified, a solid conclusion could not be drawn regarding the impact of DPP-4i on other comprehensive geriatric assessments, including BADL, CES-D, GDS, and MNA. Further studies are also needed to illustrate this link in DPP-4i users. In fact, cognitive function consists of multiple test scores, which demonstrate an effect of cognition from different aspects. A preclinical study also showed that DPP-4i treatment inhibited oxidative stress and had a beneficial effect on diabetes-related dementia, a process independent of glucose reduction [54]. This finding helps to further elucidate the impact of DPP-4i on cognitive function, which might be somewhat dependent on related neurological proteins or related signalling pathways. Indeed, vildagliptin had been reported to restore cognitive function in neurological disease models by activating BDNF signalling cascades [71].

In the current study, DPP-4i produced no stronger effect on cognitive function than other antidiabetic agents. Numerous studies have been performed to evaluate the impact of antidiabetic agents on cognitive function. For metformin, controversy still exists regarding its impact on cognitive function. In a meta-analysis involving 19 studies, no significant improvement of cognitive function or dementia was found in T2DM patients during metformin treatment [72]. However, in another prospective observational study, elderly diabetic participants receiving metformin showed a slower cognitive decline and lower dementia risk compared to nonusers [73]. Acarbose serves as an alpha-glucosidase inhibitor and is poorly absorbed after administration. This agent exerted a glucose-lowering effect only in the intestine, and its effect on cognitive function was speculated to depend on glucose control [74]. In contrast, insulin-treated participants demonstrated an improvement in the attention test, which might be associated with an increase of plasma concentrations of beta-amyloid peptide in a fasting state [75]. Thiazolidinedione (TZD) treatment with pioglitazone has also improved cognitive impairment in T2DM by activating peroxisome proliferator-activated receptor gamma (PPAR-γ) [76]. A similar improvement in the memory test was also found in patients undergoing treatment with sulfonylureas, which was linked with a reduction of glucose levels, rather than an altered insulin condition [77]. In a recent promising study evaluating the effect of antidiabetic drugs on AD, GLP-1 receptor agonists delayed neurodegenerative processes associated with T2DM [78]. For AD patients, incretin-based therapies significantly improved learning and memory ability, an action correlating with a reduction of inflammatory response and tau hyperphosphorylation [79]. SGLT2i, a novel class of antidiabetic agents, also exerted an effect on cognitive function in T2DM. According to a retrospective cohort study, SGLT2i users showed a lower incidence of dementia than DPP-4i users [16]. This effect might be attributed to a favourable lipid-soluble nature of SGLT2i, allowing them to cross the blood‒brain barrier (BBB) and exert effects on brain cognition. The noninferiority effect observed between DPP-4i and other antidiabetic agents might also result from a balance of varied results from different antidiabetic classes. Detailed head-to-head trials should be designed to strictly evaluate comparative effects of DPP-4i with other agents on cognition.

Oral DPP-4i are well tolerated and safe to use in patients with T2DM. The neuroprotective effects reported in the above studies might indirectly derive from targeting their substrates or related signalling pathways. However, available DPP-4i in the current market could not cross the BBB, which inevitably limited their maximal effects on cognitive function. Omarigliptin, a novel DPP-4i designed to solve the issues of crossing BBB, is being used to treat diabetes-related neurodegenerative diseases [80]. Further clinical trials are also needed to deeply explore the mechanism of DPP-4i treatment in modulating cognitive function.

This current meta-analysis had some strengths. Available evidence was combined to evaluate cognitive function including executive and spatial skills during DPP-4i treatment in T2DM. Compared with individual studies, this meta-analysis could provide more evidence on the effect of DPP-4i on MMSE scores. Furthermore, the impact of DPP-4i on comprehensive geriatric assessment was also demonstrated in the current study.

Nevertheless, this meta-analysis had some limitations. First, the number of identified trials was small, and no subgroup analysis on age or sex were performed. Second, identified trials had differences in size, subject characteristics, cognitive scores at baseline and DPP-4i dosage. Diabetic patients were accompanied with other diseases including stroke were pooled, which inevitably confounded the impact of DPP-4i on cognition ability. Third, this meta-analysis covered sitagliptin, vildagliptin and linagliptin, while no further analysis on other DPP-4i were compared on MMSE scores or other scores due to the limited number of studies. Although more evidence from clinical studies were included, additional data from basic research should also be recorded to complete the conclusion. This could make the reference structure more stable. Finally, analysis on side effects should also be objectively performed when cognitive function was assessed in diabetic patients during DPP-4i treatment. 

## Conclusions

Available data from clinical trials suggested that DPP-4i produced an off-target favourable effect on cognitive function in patients with T2DM. Whether these significant improvements could translate into clinical value remains to be explored with further studies involving real world data.

### Electronic supplementary material

Below is the link to the electronic supplementary material.

Supplementary Material 1

Supplementary Material 2

Supplementary Material 3

Supplementary Material 4

## Data Availability

All data generated or analysed during this study are included in this published article [and its supplementary information files].

## Abbreviations

DPP-4i: dipeptidyl peptidase-4 inhibitor
T2DM: type 2 diabetes mellitus
MMSE: Mini-Mental State Examination
BADL: Barthel Activities of Daily Living
BDNF: brain-derived neurotrophic factor
IADL: Instrumental Activities of Daily Living
GDS: Global Deterioration Scale
SGLT2i: sodium glucose cotransporter 2 inhibitors
HbA1c: glycosylated haemoglobin
RCT: randomized controlled trial
IR: insulin resistance
MNA: Mini Nutritional Assessment
TMT: Trial Making Test
VFT: Verbal Fluency Test
A&E: Attention and executive functioning
CES-D: Center for Epidemiologic Studies Depression Scale
GLP-1: glucagon-like peptide 1
LPS: lipopolysaccharide
MCI: mild cognitive impairment
AD: Alzheimer’s disease
PD: Parkinson’s disease
SDF-1α: stromal derived factor 1α
NPY: neuropeptide Y
Nrf2: NF-E2-related factor 2
BNP: brain natriuretic peptide
TZDs: thiazolidinediones
PAR2: protease-activated receptor 2
PPAR-γ: peroxisome proliferator-activated receptor gamma
Cav 1: caveolin 1
BMI: body mass index
BBB: blood‒brain barrier
WMD: weighted mean difference
CI: confidence interval
PRISMA: Preferred Reporting Items for Systematic Reviews and Meta-Analyses
M-H: Mantel-Haenszel
WOS: Web of Science
NADPH: Nicotinamide adenine dinucleotide phosphate
