# Supplementary material for: Dipeptidyl peptidase-4 inhibitors alleviate cognitive dysfunction in type 2 diabetes mellitus
Source: Lipids Health Dis. 2023 Dec 11;22:219. doi: 10.1186/s12944-023-01985-y (PMC10712048; doi:10.1186/s12944-023-01985-y)
Supplement: Supplementary file 1 — Supplementary Material 1 [file 12944_2023_1985_MOESM1_ESM.pdf]

AJE®

Editing Certificate

This document certifies that the manuscript

Dipeptidyl peptidase 4 inhibitors attenuate cognitive function in type 2 diabetes mellitus: A systematic review and meta-analysis

prepared by the authors

Feng Tao, Liu Xin

was edited for proper English language, grammar, punctuation, spelling, and overall style by one or more of the highly qualified native English speaking editors at AJE.

This certificate was issued on **August 20, 2023** and may be verified on the AJE website using the verification code **4E58-BC96-3932-98FA-28EP**.

AJE® EDITING CERTIFIED

Neither the research content nor the authors' intentions were altered in any way during the editing process. Documents receiving this certification should be English-ready for publication; however, the author has the ability to accept or reject our suggestions and changes. To verify the final AJE edited version, please visit our verification page at aje.com/certificate. If you have any questions or concerns about this edited document, please contact AJE at support@aje.com.

AJE provides a range of editing, translation, and manuscript services for researchers and publishers around the world. For more information about our company, services, and partner discounts, please visit aje.com.
