# Supplementary material for: Dipeptidyl peptidase-4 inhibitors alleviate cognitive dysfunction in type 2 diabetes mellitus
Source: Lipids Health Dis. 2023 Dec 11;22:219. doi: 10.1186/s12944-023-01985-y (PMC10712048; doi:10.1186/s12944-023-01985-y)
Supplement: Supplementary file 2 — Supplementary Material 2 [file 12944_2023_1985_MOESM2_ESM.pdf]

August 20, 2023

Dear 鑫 柳,

Thank you for choosing American Journal Experts. This manuscript, titled "Dipeptidyl peptidase 4 inhibitors alleviate cognitive dysfunction in type 2 diabetes mellitus: A systematic review and meta-analysis," is very interesting. The paper was edited for grammar, phrasing, and punctuation. In addition, many edits were made to further improve the flow and readability of the text. Below, we highlight the areas of this paper that we focused on in our edit.

Certain edits were made to remove redundant, repetitive or unnecessary phrasing and to present the information in a more straightforward manner.

Some edits were made to improve conciseness by trimming unnecessary words and streamlining the flow of your manuscript.

In cases where the meaning of the text was not clear, revisions were made to convey the information with increased clarity and reduced ambiguity.

Comments were left if further clarification would be helpful or confirmation of the meaning of the text was necessary. Please review these comments and all our changes carefully for more detailed suggestions, as well as to ensure that the final version of the manuscript is fully accurate.

Thank you again for using our editing services; we wish you the best of luck with your submission.

Best regards,

Keenan J.
Senior Editor
American Journal Experts
