# Supplementary material for: Dipeptidyl peptidase-4 inhibitors alleviate cognitive dysfunction in type 2 diabetes mellitus
Source: Lipids Health Dis. 2023 Dec 11;22:219. doi: 10.1186/s12944-023-01985-y (PMC10712048; doi:10.1186/s12944-023-01985-y)
Supplement: Supplementary file 3 — Supplementary Material 3 [file 12944_2023_1985_MOESM3_ESM.docx]

# Supplementary material

|  | **Random sequence generation** | **Allocation concealment** | **Blinding** | **Incomplete outcome** | **Selective reporting** | **Others** | **Overall** |
| --- | --- | --- | --- | --- | --- | --- | --- |
| Biessels, 2019 | Low risk | Low risk | Low risk | Unclear | Low risk | Unclear | Unclear |
| Xue，2020 | Low risk | Unclear | Unclear | Unclear | Unclear | High risk | High risk |
| Biessels, 2021 | Low risk | Low risk | Low risk | Unclear | Low risk | Unclear | Unclear |

**Supplementary Table 1.** Results of quality assessment of RCT studies using the Cochrane Collaboration’s risk-of-bias tool.

|  | **Selection** | **Comparability** | **Outcome** | **Quality Scores** |
| --- | --- | --- | --- | --- |
| Borzì，2019 | **** | * | ** | 7 |
| Isik, 2017 | **** | ** | ** | 8 |
| Bulut, 2020 | **** | ** | ** | 8 |

**Supplementary Table 2.** Results of quality assessment of non-RCT studies using the Newcastle-Ottawa Scale.


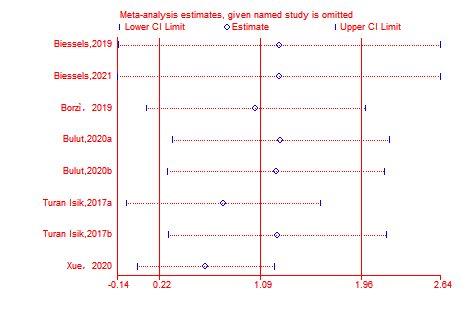


**Supplementary Fig.1.** Leave-one-out sensitivity analysis.


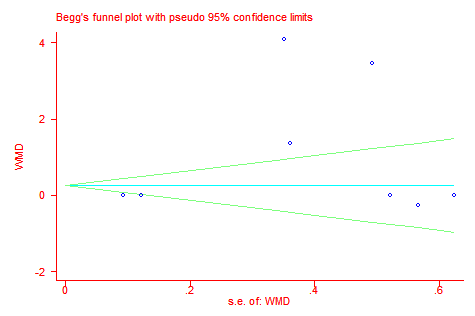


**Supplementary Fig.2.** Begg’s funnel plot for publication bias evaluation.
