# Supplementary material for: Dipeptidyl peptidase-4 inhibitors alleviate cognitive dysfunction in type 2 diabetes mellitus
Source: Lipids Health Dis. 2023 Dec 11;22:219. doi: 10.1186/s12944-023-01985-y (PMC10712048; doi:10.1186/s12944-023-01985-y)

# Search strategy

Pubmed

| Number | Query | Results |
| --- | --- | --- |
| #1 | ("Diabetes Mellitus, Type 2"[Mesh]) OR ((Diabetes Mellitus, Noninsulin-Dependent[Title/Abstract]) OR (Diabetes Mellitus, Ketosis-Resistant[Title/Abstract]) OR (Diabetes Mellitus, Ketosis Resistant[Title/Abstract]) OR (Ketosis-Resistant Diabetes Mellitus[Title/Abstract]) OR (Diabetes Mellitus, Non Insulin Dependent[Title/Abstract]) OR (Diabetes Mellitus, Non-Insulin-Dependent[Title/Abstract]) OR (Non-Insulin-Dependent Diabetes Mellitus[Title/Abstract]) OR (Diabetes Mellitus, Stable[Title/Abstract]) OR (Stable Diabetes Mellitus[Title/Abstract]) OR (Diabetes Mellitus, Type II[Title/Abstract]) OR (NIDDM[Title/Abstract]) OR (Diabetes Mellitus, Noninsulin Dependent[Title/Abstract]) OR (Diabetes Mellitus, Maturity-Onset[Title/Abstract]) OR (Diabetes Mellitus, Maturity Onset[Title/Abstract]) OR (Maturity-Onset Diabetes Mellitus[Title/Abstract]) OR (Maturity Onset Diabetes Mellitus[Title/Abstract]) OR (MODY[Title/Abstract]) OR (Diabetes Mellitus, Slow-Onset[Title/Abstract]) OR (Diabetes Mellitus, Slow Onset[Title/Abstract]) OR (Slow-Onset Diabetes Mellitus[Title/Abstract]) OR (Type 2 Diabetes Mellitus[Title/Abstract]) OR (Noninsulin-Dependent Diabetes Mellitus[Title/Abstract]) OR (Noninsulin Dependent Diabetes Mellitus[Title/Abstract]) OR (Maturity-Onset Diabetes[Title/Abstract]) OR (Diabetes, Maturity-Onset[Title/Abstract]) OR (Maturity Onset Diabetes[Title/Abstract]) OR (Type 2 Diabetes[Title/Abstract]) OR (Diabetes, Type 2[Title/Abstract]) OR (Diabetes Mellitus, Adult-Onset[Title/Abstract]) OR (Adult-Onset Diabetes Mellitus[Title/Abstract]) OR (Diabetes Mellitus, Adult Onset[Title/Abstract])) | 235756 |
| #2 | ("Dipeptidyl-Peptidase IV Inhibitors"[Mesh]) OR (Dipeptidyl Peptidase IV Inhibitors[Title/Abstract]) OR (DPP-4 Inhibitor[Title/Abstract]) OR (DPP 4 Inhibitor[Title/Abstract]) OR (Inhibitor, DPP-4[Title/Abstract]) OR (DPP-IV Inhibitor[Title/Abstract]) OR (DPP IV Inhibitor[Title/Abstract]) OR (Inhibitor, DPP-IV[Title/Abstract]) OR (DPP-4 Inhibitors[Title/Abstract]) OR (DPP 4 Inhibitors[Title/Abstract]) OR (DPP-IV Inhibitors[Title/Abstract]) OR (DPP IV Inhibitors[Title/Abstract]) OR (Gliptin[Title/Abstract]) OR (Dipeptidyl Peptidase 4 Inhibitor[Title/Abstract]) OR (Dipeptidyl-Peptidase IV Inhibitor[Title/Abstract]) OR (Dipeptidyl Peptidase IV Inhibitor[Title/Abstract]) OR (Inhibitor, Dipeptidyl-Peptidase IV[Title/Abstract]) OR (Dipeptidyl-Peptidase 4 Inhibitor[Title/Abstract]) OR (Inhibitor, Dipeptidyl-Peptidase 4[Title/Abstract]) OR (Dipeptidyl-Peptidase 4 Inhibitors[Title/Abstract]) OR (Dipeptidyl Peptidase 4 Inhibitors[Title/Abstract]) OR (Gliptins[Title/Abstract]) OR (DPP4 Inhibitor[Title/Abstract]) OR (Inhibitor, DPP4[Title/Abstract]) OR (DPP4 Inhibitors[Title/Abstract]) | 9017 |
| #3 | ("Cognition"[Mesh]) OR (Cognitions[Title/Abstract]) OR (Cognitive Function[Title/Abstract]) OR (Cognitive Functions[Title/Abstract]) OR (Function, Cognitive[Title/Abstract]) OR (Functions, Cognitive[Title/Abstract]) OR ("Cognitive dysfunction"[Mesh]) OR (Cognitive Dysfunctions[Title/Abstract]) OR (Dysfunction, Cognitive[Title/Abstract]) OR (Dysfunctions, Cognitive[Title/Abstract]) OR (Cognitive Impairments[Title/Abstract]) OR (Cognitive Impairment[Title/Abstract]) OR (Impairment, Cognitive[Title/Abstract]) OR (Impairments, Cognitive[Title/Abstract]) OR (Cognitive Disorder[Title/Abstract]) OR (Cognitive Disorders[Title/Abstract]) OR (Disorder, Cognitive[Title/Abstract]) OR (Disorders, Cognitive[Title/Abstract]) OR (Mild Cognitive Impairment[Title/Abstract]) OR (Cognitive Impairment, Mild[Title/Abstract]) OR (Cognitive Impairments, Mild[Title/Abstract]) OR (Impairment, Mild Cognitive[Title/Abstract]) OR (Impairments, Mild Cognitive[Title/Abstract]) OR (Mild Cognitive Impairments[Title/Abstract]) OR (Cognitive Decline[Title/Abstract]) OR (Cognitive Declines[Title/Abstract]) OR (Decline, Cognitive[Title/Abstract]) OR (Declines, Cognitive[Title/Abstract]) OR (Mental Deterioration[Title/Abstract]) OR (Deterioration, Mental[Title/Abstract]) OR (Deteriorations, Mental[Title/Abstract]) OR (Mental Deteriorations[Title/Abstract]) | 353224 |
| #4 | #1 AND #2 AND #3 | 55 |

Embase

| Number | Query | Results |
| --- | --- | --- |
| #1 | 'non insulin dependent diabetes mellitus'/exp OR 'adult onset diabetes':ti,ab,kw OR 'adult onset diabetes mellitus':ti,ab,kw OR 'diabetes mellitus type 2':ti,ab,kw OR 'diabetes mellitus type ii':ti,ab,kw OR 'diabetes mellitus, maturity onset':ti,ab,kw OR 'diabetes mellitus, non insulin dependent':ti,ab,kw OR 'diabetes mellitus, non-insulin-dependent':ti,ab,kw OR 'diabetes mellitus, type 2':ti,ab,kw OR 'diabetes mellitus, type ii':ti,ab,kw OR 'diabetes type 2':ti,ab,kw OR 'diabetes type ii':ti,ab,kw OR 'diabetes, adult onset':ti,ab,kw OR 'dm 2':ti,ab,kw OR 'insulin independent diabetes':ti,ab,kw OR 'insulin independent diabetes mellitus':ti,ab,kw OR 'ketosis resistant diabetes mellitus':ti,ab,kw OR 'maturity onset diabetes':ti,ab,kw OR 'maturity onset diabetes mellitus':ti,ab,kw OR 'maturity onset diabetes of the young':ti,ab,kw OR 'niddm':ti,ab,kw OR 'niddm (non insulin dependent diabetes mellitus)':ti,ab,kw OR 'non insulin dependent diabetes':ti,ab,kw OR 'non-insulin-dependent diabetes mellitus':ti,ab,kw OR 'noninsulin dependent diabetes':ti,ab,kw OR 'noninsulin dependent diabetes mellitus':ti,ab,kw OR 't2dm':ti,ab,kw OR 'type 2 diabetes':ti,ab,kw OR 'type 2 diabetes mellitus':ti,ab,kw OR 'type ii diabetes':ti,ab,kw OR 'type ii diabetes mellitus':ti,ab,kw OR 'non insulin dependent diabetes mellitus':ti,ab,kw | 402302 |
| #2 | 'cognition'/exp OR 'cognitive accessibility':ti,ab,kw OR 'cognitive balance':ti,ab,kw OR 'cognitive dissonance':ti,ab,kw OR 'cognitive function':ti,ab,kw OR 'cognitive structure':ti,ab,kw OR 'cognitive symptoms':ti,ab,kw OR 'cognitive task':ti,ab,kw OR 'cognitive thinking':ti,ab,kw OR 'neurobehavioural manifestations':ti,ab,kw OR 'volition':ti,ab,kw OR 'cognition':ti,ab,kw OR 'cognitive defect'/exp OR 'cognition disorder':ti,ab,kw OR 'cognition disorders':ti,ab,kw OR 'cognitive defects':ti,ab,kw OR 'cognitive deficit':ti,ab,kw OR 'cognitive disability':ti,ab,kw OR 'cognitive disorder':ti,ab,kw OR 'cognitive disorders':ti,ab,kw OR 'cognitive dysfunction':ti,ab,kw OR 'cognitive impairment':ti,ab,kw OR 'delirium, dementia, amnestic, cognitive disorders':ti,ab,kw OR 'overinclusion':ti,ab,kw OR 'response interference':ti,ab,kw OR 'cognitive defect':ti,ab,kw | 3540871 |
| #3 | 'dipeptidyl peptidase iv inhibitor'/exp OR 'dipeptidyl peptidase 4 inhibitor':ti,ab,kw OR 'dipeptidyl peptidase iv inhibitors':ti,ab,kw OR 'dipeptidyl-peptidase iv inhibitors':ti,ab,kw OR 'dipeptidylpeptidase 4 inhibitor':ti,ab,kw OR 'dipeptidylpeptidase iv inhibitor':ti,ab,kw OR 'dpp 4 inhibitor':ti,ab,kw OR 'dpp iv inhibitor':ti,ab,kw OR 'gliptin':ti,ab,kw OR 'gliptins':ti,ab,kw OR 'dipeptidyl peptidase iv inhibitor':ti,ab,kw | 27953 |
| #4 | #1 AND #2 AND #3 | 1133 |

WOS

| Number | Query | Results |
| --- | --- | --- |
| #1 | TS=(Diabetes melitus,type 2 OR Diabetes Mellitus, Noninsulin-Dependent OR Diabetes Mellitus, Ketosis-Resistant OR Diabetes Mellitus, Ketosis Resistant OR Ketosis-Resistant Diabetes Mellitus OR Diabetes Mellitus, Non Insulin Dependent OR Diabetes Mellitus, Non-Insulin-Dependent OR Non-Insulin-Dependent Diabetes Mellitus OR Diabetes Mellitus, Stable OR Stable Diabetes Mellitus OR Diabetes Mellitus, Type II OR NIDDM OR Diabetes Mellitus, Noninsulin Dependent OR Diabetes Mellitus, Maturity-Onset OR Diabetes Mellitus, Maturity Onset OR Maturity-Onset Diabetes Mellitus OR Maturity Onset Diabetes Mellitus OR MODY OR Diabetes Mellitus, Slow-Onset OR Diabetes Mellitus, Slow Onset OR Slow-Onset Diabetes Mellitus OR Type 2 Diabetes Mellitus OR Noninsulin-Dependent Diabetes Mellitus OR Noninsulin Dependent Diabetes Mellitus OR Maturity-Onset Diabetes OR Diabetes, Maturity-Onset OR Maturity Onset Diabetes OR Type 2 Diabetes OR Diabetes, Type 2 OR Diabetes Mellitus, Adult-Onset OR Adult-Onset Diabetes Mellitus OR Diabetes Mellitus, Adult Onset) and Preprint Citation Index | 459747 |
| #2 | TS=(Dipeptidyl-Peptidase IV Inhibitors OR Dipeptidyl Peptidase IV Inhibitors OR DPP-4 Inhibitor OR DPP 4 Inhibitor OR Inhibitor, DPP-4 OR DPP-IV Inhibitor OR DPP IV Inhibitor OR Inhibitor, DPP-IV OR DPP-4 Inhibitors OR DPP 4 Inhibitors OR DPP-IV Inhibitors OR DPP IV Inhibitors OR Gliptin OR Dipeptidyl Peptidase 4 Inhibitor OR Dipeptidyl-Peptidase IV Inhibitor OR Dipeptidyl Peptidase IV Inhibitor OR Inhibitor, Dipeptidyl-Peptidase IV OR Dipeptidyl-Peptidase 4 Inhibitor OR Inhibitor, Dipeptidyl-Peptidase 4 OR Dipeptidyl-Peptidase 4 Inhibitors OR Dipeptidyl Peptidase 4 Inhibitors OR Gliptins OR DPP4 Inhibitor OR Inhibitor, DPP4 OR DPP4 Inhibitors) and Preprint Citation Index | 15143 |
| #3 | TS=(Cognition OR Cognitions OR Cognitive Function OR Cognitive Functions OR Function, Cognitive OR Functions, Cognitive OR Cognitive dysfunction OR Cognitive Dysfunctions OR Dysfunction, Cognitive OR Dysfunctions, Cognitive OR Cognitive Impairments OR Cognitive Impairment OR Impairment, Cognitive OR Impairments, Cognitive OR Cognitive Disorder OR Cognitive Disorders OR Disorder, Cognitive OR Disorders, Cognitive OR Mild Cognitive Impairment OR Cognitive Impairment, Mild OR Cognitive Impairments, Mild OR Impairment, Mild Cognitive OR Impairments, Mild Cognitive OR Mild Cognitive Impairments OR Cognitive Decline OR Cognitive Declines OR Decline, Cognitive OR Declines, Cognitive OR Mental Deterioration OR Deterioration, Mental OR Deteriorations, Mental OR Mental Deteriorations) and Preprint Citation Index | 873676 |
| #4 | #1 AND #2 AND #3 | 148 |

Cochrane library


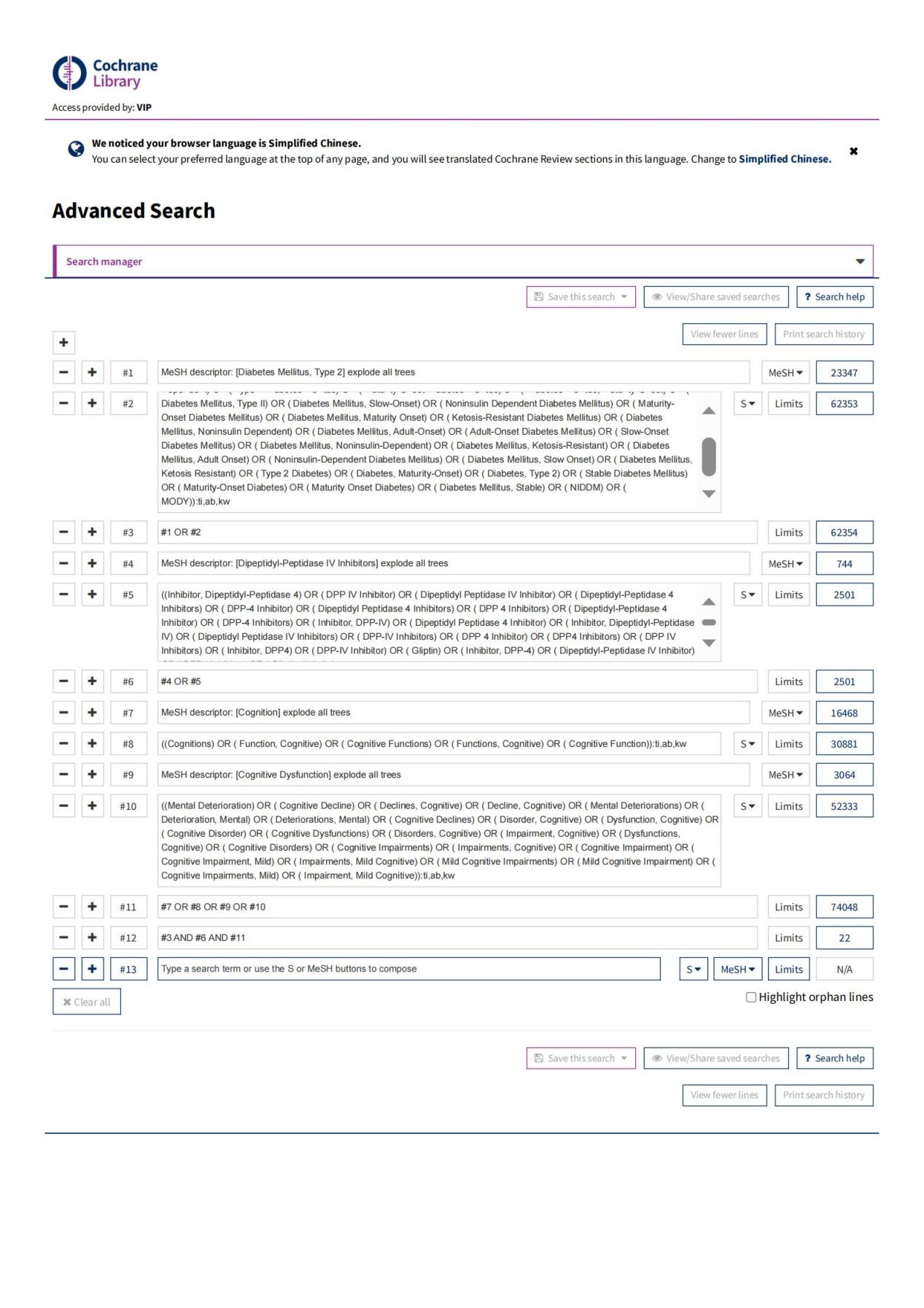


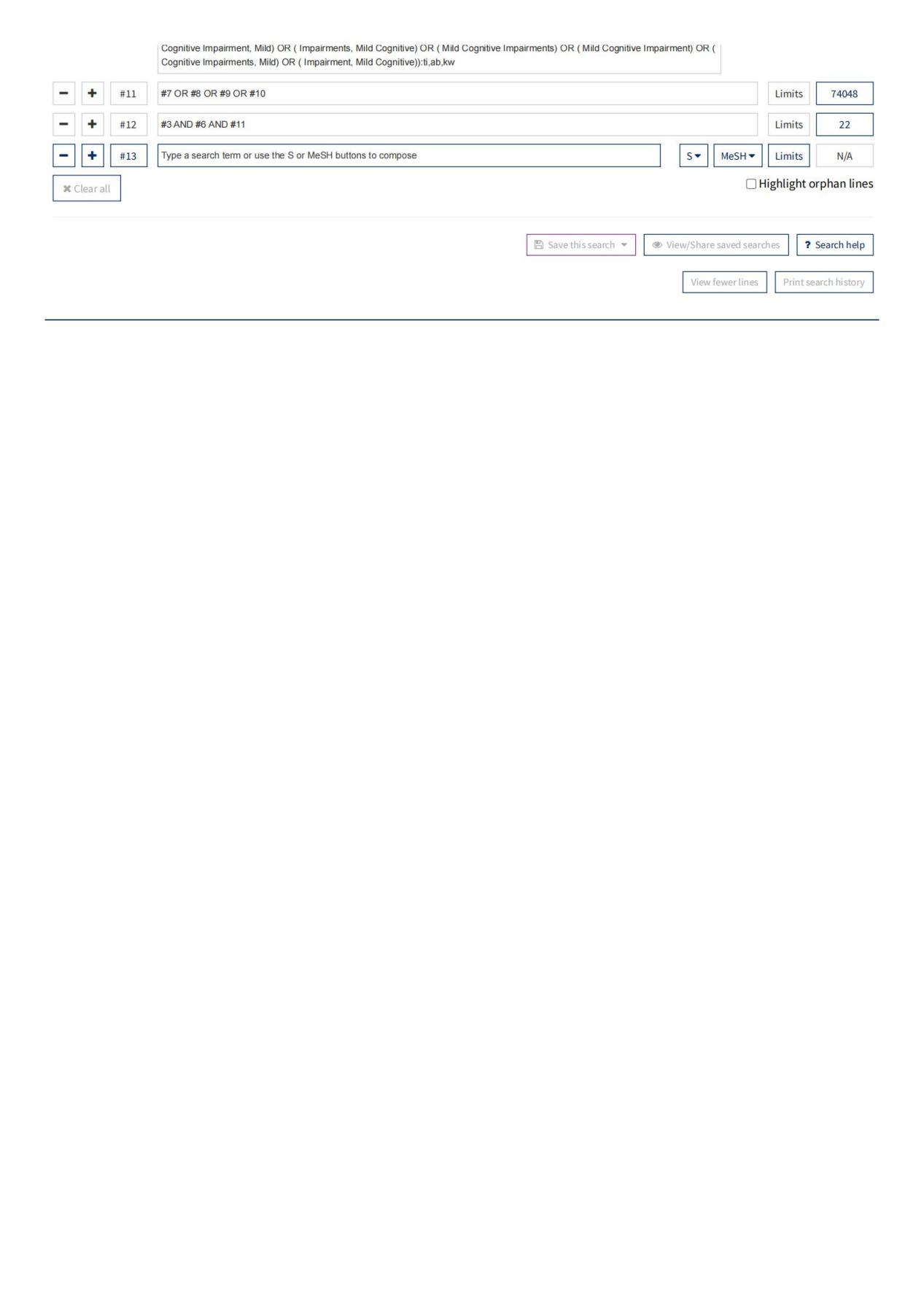

Supplement: Supplementary file 4 — Supplementary Material 4 [file 12944_2023_1985_MOESM4_ESM.docx]
